# Male mouse skeletal muscle lacking HuR shows enhanced glucose disposal at a young age

**DOI:** 10.3389/fphys.2024.1468369

**Published:** 2025-02-19

**Authors:** Robert C. Noland, Sujoy Ghosh, Carlos J. Crisanto, Antonio Aleman, McKenna K. Chaney, Maitri K. Chauhan, Layla G. Loftis, Ally C. Goad, Christin F. Rickman, Samuel E. Velasquez, Jaycob D. Warfel

**Affiliations:** ^1^ Pennington Biomedical Research Center, Baton Rouge, LA, United States; ^2^ Skeletal Muscle Metabolism for RCN, and Functional Genomics for SG, Baton Rouge, LA, United States; ^3^ Biology Department, Christian Brothers University, Memphis, TN, United States; ^4^ Department of Biological Sciences, Southeastern Louisiana University, Hammond, LA, United States; ^5^ Department of Biological Sciences, The University of Tennessee at Martin, Martin, TN, United States

**Keywords:** human antigen R, metabolic flexibility, RNA-binding proteins, fat oxidation, carbohydrate oxidation

## Abstract

**Introduction:**

Metabolic flexibility is the ability of a system to switch between metabolic substrates. Human and murine skeletal muscle tissues and cells with decreased activity of the regulatory RNA-binding protein, human antigen R (HuR), have decreased capacity for fat oxidation, and thus decreased metabolic flexibility. In this study, we aimed to assess the preference for carbohydrates in mice lacking HuR in skeletal muscle.

**Methods:**

Experiments were performed on weight-matched control and HuR knockout mice of both sexes. Palmitate and pyruvate oxidation were performed in mouse muscle following the release of ^14^CO_2_. *In vivo* glucose and lipid uptake were assayed in mouse tissue following nonmetabolizable ^3^H-2-deoxyglucose or ^14^C-bromopalmitate injection. Transcriptomic analyses were performed in the skeletal muscle of all mice, followed by qPCR validation of select genes. Serum lactate and glucose levels were measured in mice *via* tail nick, and the muscle glycogen level was measured through colorimetric assay. Indirect calorimetry was used to measure respiratory exchange ratios.

**Results:**

Male muscle-specific HuR knockout mice showed increased glucose uptake relative to controls, specifically in skeletal muscle, and have increased muscle glycogen content. These mice also displayed greater respiratory exchange ratios than controls. None of these differences were noted in females. Transcriptomics showed far more differences between male and female mice than between control and HuR knockout mice. However, differential gene expression between male and female mice was diminished by 50% following the removal of HuR. Male HuR knockout mouse skeletal muscle had increased glycolytic gene expression relative to controls but showed no difference relative to females of the same genotype. Both palmitate and pyruvate oxidation were decreased in the skeletal muscle of male HuR knockout mice relative to controls, and serum lactate levels were increased. No notable differences were seen in females between genotypes.

**Discussion:**

The increase in the markers of glucose utilization with decreased HuR activity in male mice may indicate a switch toward glycolysis as compensation for decreased fat oxidation. These results continue to highlight a sex dependence on HuR as a driver of fat oxidation in mouse skeletal muscle while also indicating that muscle itself shows greater ambiguity between males and females following the removal of HuR.

## 1 Introduction

Biological systems are metabolically flexible, allowing them to switch between different nutrient substrates for energy production. Flexibility can vary, and the degree to which a system can switch between different fuels when available has noteworthy implications in metabolic disease pathology (Goodpaster and Sparks, 2017; Storlien et al., 2004). As ectopic accumulation of lipids is known to interfere with insulin signaling (Goodpaster and Kelley, 2002; Samuel and Shulman, 2016), a decreased capacity to switch to lipid oxidation can be particularly detrimental. Humans with a decreased ability to adapt to a high-fat diet show an increased tendency toward fat mass gain, which is known to contribute to the development of metabolic disease (Begaye et al., 2020).

We have shown that the RNA-binding protein and master regulator, human antigen R (HuR), is important for the control of metabolic flexibility in skeletal muscle through its promotion of fatty acid oxidation in both mice and humans (Mynatt et al., 2019; Stone et al., 2021). However, this is a sex-specific phenomenon as only male mice experience a decrease in fatty acid oxidation in skeletal muscle when HuR is removed. This decreased fatty acid oxidation is accompanied by increased fat mass gain, which leads to more rapid development of hallmarks of insulin resistance relative to controls. Data on female HuR skeletal muscle knockout (HuR^m−/−^) mice are of particular interest as these mice have increased fat mass relative to controls but show no decrease in skeletal muscle fatty acid oxidation capacity and trend toward enhanced glucose clearance relative to controls (Stone et al., 2021).

Humans with decreased metabolic flexibility have decreased levels of transcripts regulated by HuR in skeletal muscle, regardless of whether the subjects are male or female (Mynatt et al., 2019), and compelling evidence shows that humans with decreased metabolic flexibility also show increases in serum lactate levels following exercise training (San-Millan and Brooks, 2018). Additionally, several studies have shown that male HuR^m−/−^ mice consistently show an increased respiratory exchange ratio (RER) relative to controls (Mynatt et al., 2019; Stone et al., 2021; Janice Sanchez et al., 2019). These studies, coupled with the increased glucose clearance observed in female HuR^m−/−^ mice, could suggest an increased reliance upon glycolysis within systems with decreased metabolic flexibility.

We hypothesized that HuR^m−/−^ mice may rely more heavily on glucose and that insulin signaling is interfered with in male HuR^m−/−^ mice as ectopic lipid content increases. To evaluate this hypothesis, we weight matched HuR^m−/−^ and control mice and tested several physiological parameters of glucose and lipid utilization. These assays included transcriptomic analyses, skeletal muscle pyruvate and palmitate oxidation assays, *in vivo* glucose and palmitate uptake assays, and glucose tolerance tests.

## 2 Materials and methods

### 2.1 Animals

The breeding of skeletal muscle-specific HuR-deficient (HuR^m−/−^) mice and littermate controls (HuR^fl/fl^) has been previously described (Mynatt et al., 2019). Briefly, mice floxed at *elavl1*, the gene encoding HuR, were purchased from the Jackson Laboratory (Ghosh et al., 2009) (Stock # 021431) and bred to mice expressing Cre recombinase under the control of the Mlc1f promoter (Bothe et al., 2000) (Jackson Laboratory, Stock # 024713) to delete HuR in skeletal muscle. All mice were on a C57BL/6 background. Mice were bred and group-housed at room temperature (RT; 22°C–24°C) under a 12:12-h light:dark cycle and allowed *ad libitum* access to food (Purina Mills 5015) and water. Mice were either 10–12 or 20–24 weeks of age when euthanized and the age of each group is listed for each dataset. The mice were euthanized by cervical dislocation, and tissues were collected, snap-frozen in liquid nitrogen, and stored at −80°C until subsequent analyses could be performed. Mixed gastrocnemius skeletal muscle was powdered and used for all assays presented, unless otherwise noted. The mice were euthanized according to the approved procedures of the Panel on Euthanasia of the American Veterinary Medical Association.

### 2.2 Animal procedures

Body composition was measured using a Bruker NMR Minispec (Bruker Corporation, Billerica, MA, United States). Glucose tolerance tests and blood lactate measurements were performed after a 4-h fast as previously described (Warfel et al., 2017). Briefly, at 10–12 weeks of age after measuring baseline blood glucose and lactate levels via the tail vein using either a lactate plus meter (Nova Biomedical) or a blood glucose monitoring system (OneTouch Ultra 2), mice received a 0.2-mL intraperitoneal injection of 20% D-glucose (40 mg glucose per mouse; 1.5 g/kg for males and 2 g/kg for females), and blood glucose levels were subsequently monitored at 20 min, 40 min, and 60 min post-injection. Behavioral and indirect calorimetry studies were done in a 16-chamber Promethion system (Sable Systems International, Las Vegas, NV, United States) on the mice at 20–22 weeks of age. For these experiments, the mice were single-housed at RT (22°C–24°C) under a 12:12-h light:dark cycle and allowed *ad libitum* access to food and water. Gastrocnemius muscle was used for the measurement of the total glycogen content in HuR^m−/−^ and control mice. A glycogen assay kit (Abcam, ab65620) was used for glycogen measurement following the manufacturer’s instructions.

### 2.3 RNA isolation

RNA was extracted from 20 to 30 mg of powdered mouse tissue using TRIzol (Thermo Fisher Scientific, Waltham, MA, United States), as previously described (Lam et al., 2014). Briefly, samples were homogenized in 300 μL TRIzol and allowed to sit at RT for 5 min, and then, 0.2 mL of chloroform was added. The samples were shaken vigorously for 15 s and allowed to sit at RT for 2–3 min before they were centrifuged (12,000 × g; 15 min; 4°C) to induce phase separation. Approximately 150 µL of the upper aqueous supernatant containing RNA was transferred to a new microcentrifuge tube to which 150 µL of 70% ethanol was added, and the samples were vortexed. RNA was then isolated using an RNeasy kit (QIAGEN, Valencia, CA, United States) with DNAse treatment, as per the manufacturer’s instructions. RNA content and quality (260/280 ratio range 1.9–2.1) were assessed using NanoDrop 1000.

### 2.4 RNA sequencing

Whole gastrocnemius muscle of HuR^m−/−^ and control mice (N = 5–7 per group) were used for total RNA extraction, as described in Section 2.3. RNA-seq libraries were constructed using Illumina’s TruSeq Stranded Total RNA Library Prep Kit with Ribo-Zero. RNA was sequenced on the Illumina NextSeq 500 using the High Output v2 Kit and paired-end sequencing forward and reverse reads (2 × 75 bp) with 75 million reads/sample. Gene-level aggregated raw counts were normalized via the TMM algorithm in edgeR (Robinson et al., 2010), and subsequent differential gene expression analysis was conducted via limma (Ritchie et al., 2015). Significantly regulated genes were defined as genes with an adjusted *p*-value of differential gene expression (false discovery rate or FDR) ≤ 0.05. Biological pathway enrichment was evaluated via Gene Set Enrichment Analysis (GSEA) (Mootha et al., 2003) with pathways drawn from the Kyoto Encyclopedia of Genes and Genomes (KEGG), available from Molecular Signatures Database, MSigDB (Liberzon et al., 2011), from WikiPathways (Agrawal et al., 2024), or Gene Ontology and Biological Pathways (GOBP) (Nguyen et al., 2015). For pathway analysis, pathways with an adjusted enrichment *p*-value (FDR) ≤ 0.1 were considered to be significantly regulated, following established methods (Griss et al., 2020; Reimand et al., 2019).

### 2.5 Quantitative RT-PCR

Total RNA from tissues was isolated as described above. cDNA was then synthesized using an iScript cDNA synthesis kit and used for qRT-PCR with the SYBR Green system (Bio-Rad, Hercules, CA, United States). Analysis was conducted using the Norma-gene macro, as previously described (Heckmann et al., 2011; O’Connell et al., 2017). The mouse *ppib* transcript was included as an additional reference gene within the Norma-gene macro to provide additional power. Primer details are given in Supplementary Table S1.

### 2.6 Substrate oxidation assays

Mixed gastrocnemius muscle homogenates were prepared as previously described [14]. Fatty acid oxidation was measured as the liberation of ^14^CO_2_ from [1–^14^C]-palmitate (ARC, St. Louis MO). Oxidation of palmitate at a final concentration of 200 μM unlabeled and 0.625 nCi/μL labeled was measured over the course of 30 min. Homogenates were incubated with or without 1 mM unlabeled pyruvate to measure the inhibition of palmitate oxidation. Pyruvate oxidation was measured using identical procedures, with the exception of using a final concentration of 1 mM pyruvate unlabeled and 0.625 nCi/μL [3–^14^C]-pyruvate (ARC, St. Louis MO) ± unlabeled palmitate (200 µM) to assess substrate competition. All reactions were conducted using media (pH 7.4) consisting of 100 mM sucrose, 60 mM EDTA, 10 mM Tris HCl, 10 mM K_2_HPO_4_, 80 mM KCl, 1 mM MgCl_2_ 6H_2_O, 1 mM L-carnitine, 0.05 mM malate, 1 mM DTT, 0.05 mM nicotinamide adenine dinucleotide, 2 mM ATP, and 0.05 mM CoA.

### 2.7 Glucose and lipid uptake assays

Using previously described procedures (Hom et al., 1984) with modifications, at 10–12 weeks of age, HuR^m−/−^ and control mice were fasted for 4 h and injected with either 200 µL of 20% glucose supplemented with 25 µL of 1 mCi/mL deoxy-D-glucose, 2-[1–^3^H] (ARC), or oral gavaged with 200 μL of olive oil supplemented with 125 μL 0.1 mCi/mL S-2-bromopalmitic acid [1–^14^C] (ARC). For the preparation of the palmitic acid solution, the stock solution was primarily ethanol, which was evaporated with compressed air prior to the addition of olive oil. Glucose-injected mice were euthanized after 40 min and palmitate-gavaged mice were euthanized after 3 h. Tissues were collected following euthanasia, and up to 75 mg of each tissue was placed in 1.5 mL of hexadecyltrimethylammonium bromide (Sigma) containing solubilization buffer and incubated for 6 h in a 65-C water bath with gentle shaking. Then, 10 mL of scintillation cocktail was added to each vial, and the vials were read for total ^3^H and ^14^C counts, respectively, in a scintillation counter.

### 2.8 Study approval

Animal studies were conducted at the Pennington Biomedical Research Center’s AALAC-approved facility. All experiments were in compliance with the NIH Guide for the Care and Use of Laboratory Animals and approved by the Pennington Biomedical Research Center Institutional Animal Care and Use Committee under PBRC IACUC Protocol #1049.

### 2.9 Statistics

Data are expressed as the mean ± SEM. For single-variable comparisons between control HuR^m−/−^ mice or between males and females, GraphPad Prism software was used to determine significant differences with paired, equal-variance two-tailed Student's *t*-tests, where normality was established using the D’Agostino–Pearson normality test. For multiple comparisons between genotypes and sex groups, GraphPad Prism software was used to determine significant differences with two-way ANOVA using Tukey’s *post hoc* analysis. For either test, *p* < 0.05 was considered significant.

## 3 Results

### 3.1 HuR^m−/−^ male mice clear glucose better than controls at a young age

**FIGURE 1:**
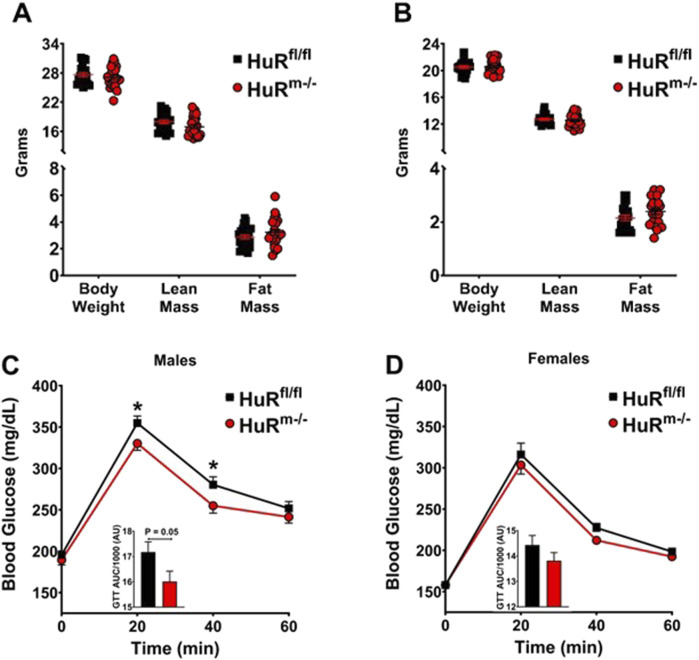
Male HuR^m−/−^ mice show increased glucose clearance relative to controls when weight-matched. **(A, B)** Body weight, lean mass, and fat mass are shown for control (black) and HuR^m−/−^ (red) males **(A)** and females **(B)**. **(C, D)** GTT are shown with the area under the curve displayed as an inset figure for control (black) and HuR^m−/−^ (red) males **(C)** and females **(D)**. N = 21–22; *p ≤ 0.05.

We previously reported that 20- to 24-week-old HuR^m−/−^ male mice have increased fat mass and impaired glucose clearance (Mynatt et al., 2019; Stone et al., 2021). As adiposity is strongly linked to glucose intolerance (Goodpaster and Sparks, 2017; Goodpaster and Kelley, 2002), we sought to determine whether glucose intolerance preceded the development of adiposity. In the present study, 10- to 12-week-old HuR^m−/−^ male mice exhibited similar levels of body weight and fat mass as age-matched controls (Figure 1A). Interestingly, in the absence of differences in adiposity, young HuR^m−/−^ male mice exhibited improved glucose tolerance (Figure 1C). In addition, our previous work reported sex-specific differences in HuR^m−/−^ mice. Specifically, while 20- to 24-week-old female HuR^m−/−^ mice exhibited similar increases in adiposity as HuR^m−/−^ male mice, unlike their male counterparts, females had improved glucose clearance rates (Stone et al., 2021). Within the present study, we tested the link between adiposity and glucose tolerance in 10- to 12-week-old mice, which showed similar body weight and fat mass between HuR^m−/−^ vs. floxed controls (Figure 1B). Surprisingly, the results herein suggest that in the absence of expansion of adiposity, female HuR^M−/−^ mice do not exhibit improvements in glucose tolerance (Figure 1D).

### 3.2 Enhanced glucose clearance in HuR^m−/−^ male mice is seen principally in skeletal muscle

**FIGURE 2:**
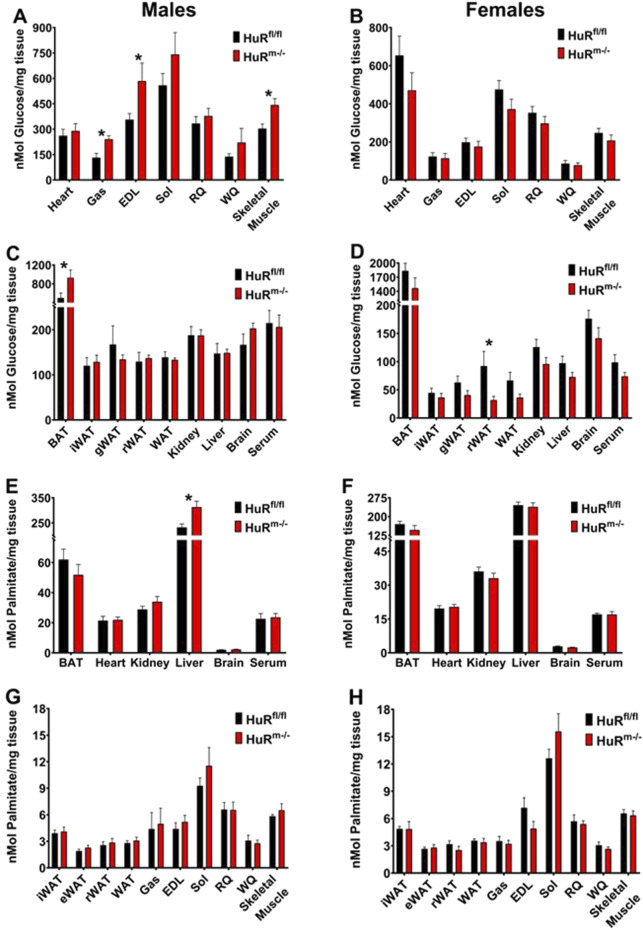
Increased glucose uptake is localized primarily to skeletal muscle in male HuR^m−/−^ mice. **(A–D)** Glucose uptake is shown in muscle **(A, B)** and additional **(C, D)** tissues from control (black) and HuR^m−/−^ (red) males **(A, C)** and females **(B, D)**. **(E–H)** Palmitate uptake is shown in various tissues **(E, F)** and skeletal muscle and white adipose tissues **(G, H)** from control (black) and HuR^m−/−^ (red) males **(E, G)** and females **(F, H)**. BAT, brown adipose tissue; iWAT, inguinal white adipose tissue; gWAT, gonadal white adipose tissue; Gas, gastrocnemius; EDL, extensor digitorum longus; Sol, soleus; RQ, red quadriceps; WQ, white quadriceps; muscle, average of all muscles. N = 9–10; *p ≤ 0.05.

To determine where glucose clearance may be enhanced in male HuR^m−/−^ mice, we utilized these same 10- to 12-week-old mice to monitor glucose uptake following injection with 1,2-^3^H, 2-deoxy-D-glucose (2DG). Male HuR^m−/−^ mice show a significant increase in glucose uptake in skeletal muscle when all tissues are averaged together as a whole, and this increase is especially localized to tissues with a significant proportion of type II skeletal muscle fibers such as gastrocnemius and extensor digitorum longus (EDL) (Figures 2A, C). Females on the other hand show no increases in uptake in any measured tissues but have a slightly decreased clearance in adipose tissue (Figures 2B, D). The only other tissue where significant differences in glucose uptake were noted was brown adipose tissue (BAT), where glucose uptake is significantly higher in HuR^m−/−^ males than in controls. Given the common origin of BAT and skeletal muscle as Myf5^+^ cells (Jung et al., 2019), we verified that mRNA of *elavl1*, the gene encoding HuR, was not expressed at a lower level in BAT of HuR^m−/−^ animals as it was it gastrocnemius compared to controls (Supplementary Figure S1). Although the result of increased glucose uptake in BAT in the absence of skeletal muscle HuR is intriguing and warrants further investigation, we did not perform further analyses of this phenomenon.

In addition to monitoring glucose uptake, we also monitored tissue uptake of 1-^14^C-S-2-bromopalmitate (2BP) to determine whether baseline clearances in dietary fat contributed to enhanced fat mass gain. HuR^m−/−^ males exhibited significantly increased uptake of 2BP only in the liver (Figures 2E, G), whereas no differences in lipid uptake were observed between genotypes in female mice (Figures 2F, H).

### 3.3 HuR^m−/−^ male mice have an increased glycolytic mRNA signature in skeletal muscle

**FIGURE 3:**
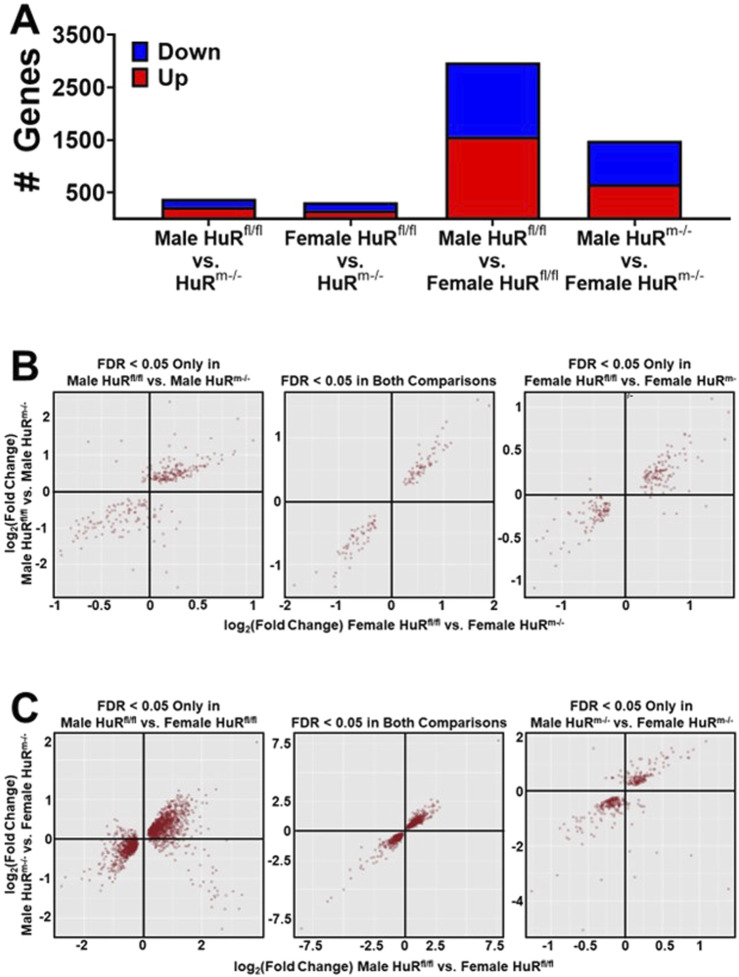
Removal of HuR from skeletal muscle reduces differential gene expression between males and females by half. **(A)** Number of differentially expressed genes between control and HuR^m−/−^ mice separated by sex, or male and female mice separated by genotype. **(B)** Scatterplot analyses of log_2_ (fold change) between females of different genotypes versus males of different genotypes. First panel plots genes that are significantly differentially expressed (FDR<0.05) between male animals of different genotypes only. Middle panel plots genes that are significantly differentially expressed (FDR<0.05) between male and female animals of different genotypes. Last panel plots genes that are significantly differentially expressed (FDR<0.05) between female animals of different genotypes only. **(C)** Scatterplot analyses plotting log_2_ (fold change) between sexes of controls versus sexes of HuR^m−/−^ mice. First panel plots genes that are significantly differentially expressed (FDR<0.05) between sexes in controls only. Middle panel plots genes that are significantly differentially expressed (FDR<0.05) between sexes within both genotypes. Last panel plots genes that are significantly differentially expressed (FDR<0.05) between sexes in HuR^m−/−^ mice only.

A second cohort of mice of 20 weeks of age (WOA) was studied for better comparison to previous studies (Mynatt et al., 2019; Stone et al., 2021). Mice from each genotype controlled for weight were used for transcriptomics analyses conducted on total RNA isolated from skeletal muscle of HuR^m−/−^ and control mice. We utilized gastrocnemius muscle in order to represent a sample known to contain a relatively equal number of type 1 and type 2 muscle fibers (Schiaffino and Reggiani, 2011), which also showed enhanced glucose clearance during uptake assays (Figure 2A). Reporting only genes with an FDR < 0.05, we found that the total number of differentially expressed genes between male HuR^m−/−^ and control samples was 384, whereas female mice had 315 differentially expressed genes (Figure 3A). Differences between male and female skeletal muscle were much more pronounced, with 2,974 genes being differentially expressed between control male and female samples. Remarkably, the removal of HuR decreased this number by 50% to 1,483 differentially expressed genes.

Scatterplots were generated with genes at FDR<0.05 by comparing Log_2_ (fold change) between all groups. These comparisons were of genes that were differentially expressed in either the male HuR^fl/fl^ vs. male HuR^m−/−^ comparison (left panel, Figure 3B), female HuR^fl/fl^ vs. female HuR^m−/−^ comparison (right panel, Figure 3B), or differentially expressed in both comparisons (middle panel, Figure 3B). These plots reveal that among both common and uniquely differentially expressed genes between groups, the direction of change in expression in HuR^m−/−^ mice compared to controls is largely consistent regardless of sex. When similar scatterplots are created comparing sexes within a given genotype (Figure 3C), the direction of change in female mice tends to be consistent compared to males regardless of the genotype. Collectively, these results demonstrate that the removal of HuR results in few changes in gene expression relative to controls but greatly diminishes transcriptomic differences between male and female gastrocnemius muscle.

Through pathway enrichment analysis *via* GSEA, we determined pathways that were significantly different in group comparisons with FDR ≤ 0.1. Of the top 60 pathways identified (Supplementary Figure S2A), the Ppar signaling pathway was number 3, and the glycolysis/gluconeogenesis pathway was number 7. The Ppar signaling pathway is a known regulator of both fat metabolism and lipid biosynthesis (Christofides et al., 2021), and we have previously reported differences in expression levels of genes within this pathway between male HuR^m−/−^ and control male mice (Stone et al., 2021). The differential expression in Ppar signaling pathway genes thus likely relates to metabolic changes within skeletal muscle lacking HuR (Supplementary Figure S3).

**FIGURE 4:**
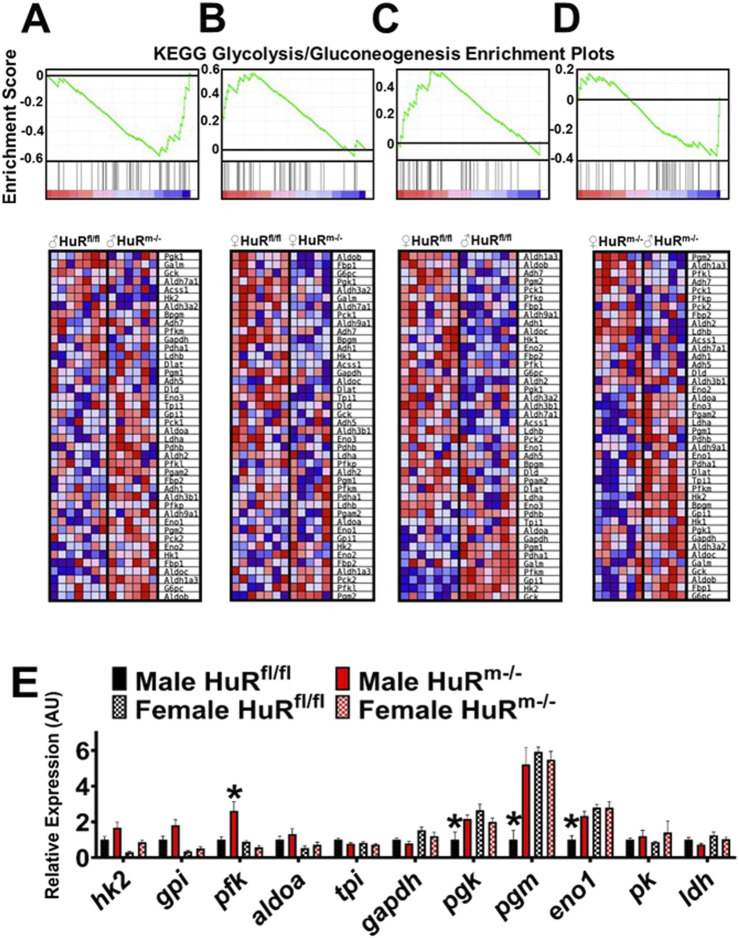
Male HuR^m−/−^ mice show an increased glycolytic transcript signature. **(A–D)** KEGG enrichment plots and Gene Set Enrichment Analyses showing differential expression of glycolysis/gluconeogenesis genes for each comparison of genotypes or sexes. **(A)** Male genotype comparison (downregulated in controls, FDR = 0.08). **(B)** Female genotype comparison (upregulated in controls, FDR = 0.03). **(C)** Control sex comparison (upregulated in females, FDR = 0.08). **(D)** HuR^m−/−^ sex comparison (not significant, FDR = 0.68). **(E)** qPCR analysis of glycolytic genes for control (black) and HuR^m−/−^ (red) males (solid) and females (checkered). N = 7–8; *p ≤ 0.05 for the marked bar compared to all other groups.

Further analysis of differences in the KEGG pathway termed “glycolysis/gluconeogenesis” between groups (Figures 4A–D) indicated differences that in general followed the same trend as was found in the Ppar signaling pathway. Significant enrichment was found in female controls relative to male controls with FDR = 0.08 and in female controls relative to female HuR^m−/−^ with FDR = 0.03. In contrast to females, male HuR^m−/−^ gastrocnemius muscle showed enrichment in glycolysis/gluconeogenesis pathway gene expression with FDR = 0.08. Strikingly, the pathway enrichment found in female relative to control males was not seen between HuR^m−/−^ males and females (FDR = 0.68).

To further confirm these results indicating that female muscle has enhanced glycolytic gene expression relative to males, which is ablated in the absence of HuR, we used qPCR to analyze the expression of glycolytic genes within the four groups (Figure 4E). Significance calculations using two-way ANOVA suggest that 3 of the 11 genes measured are significantly decreased in expression in control male muscle relative to females, whereas only 1 gene, *pfk*, is significantly different between males and females of the HuR^m−/−^ genotype, and this is due to an increase in this gene in male over female levels. Whereas no genes are significantly different between females of different genotypes, skeletal muscle HuR knockout results in increased expression of four genes in HuR^m−/−^ males relative to controls. Specifically, for *pgk*, *pgm*, and *eno1*, knockout of HuR from skeletal muscle of males renders expression levels much more similar to those in female control and HuR^m−/−^ muscle. These results reinforce the suggestion that the knockout of HuR from skeletal muscle results in a more similar gene expression pattern between male and female gastrocnemius, including an increased glycolytic signature in males that is more similar to that seen in female controls.

### 3.4 HuR^m−/−^ male mice show additional markers of increased carbohydrate usage such as increased RER, serum lactate, and muscle glycogen levels

**FIGURE 5:**
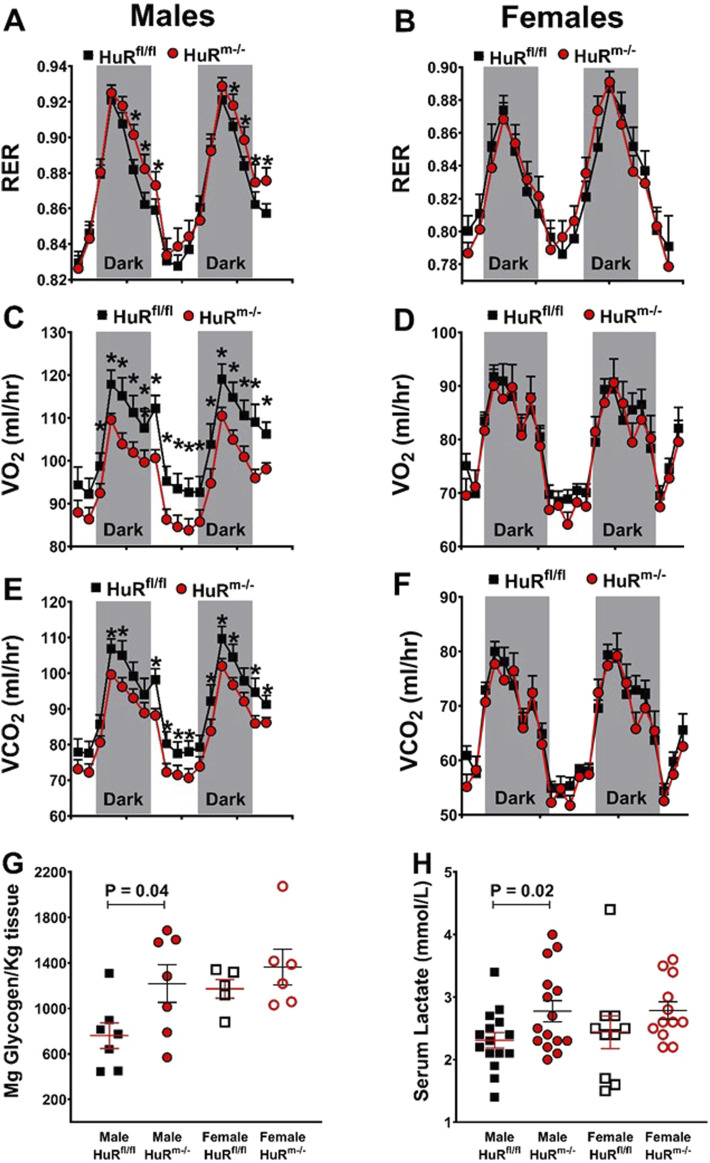
Male HuR^m−/−^ mice show an increased respiratory exchange ratio and muscle glycogen and serum lactate levels. **(A, B)** RERs are plotted for control (black) and HuR^m−/−^ (red) males **(A)** and females **(B)**. Total oxygen consumption is plotted for control (black) and HuR^m−/−^ (red) males **(C)** and females **(D)**. Total carbon dioxide production is plotted for control (black) and HuR^m−/−^ (red) males **(E)** and females **(F)**. **(G)** Gastrocnemius glycogen content is plotted for control (black) and HuR^m−/−^ (red) males (closed) and females (open). **(H)** Serum lactate levels are plotted for control (black) and HuR^m−/−^ (red) males (closed) and females (open). N = 5–8; *p ≤ 0.05.

In addition to the mRNA analyses above, we used indirect calorimetry to assess metabolic parameters for HuR^m−/−^ mice and weight-matched controls. As with previous results (Mynatt et al., 2019; Stone et al., 2021), we found increases in respiratory exchange ratios (RERs) for male but not female HuR^m−/−^ mice relative to controls (Figures 5A, B). Increases in RERs in male HuR^m−/−^ mice are reflective of decreases in oxygen consumption that are more robust than decreases in carbon dioxide production (Figures 5C–F). Although RER can be influenced by total activity and food intake, we did not find any differences in these parameters between groups (Supplementary Figure S4).

We then tested muscle and serum for glycogen content and lactate, respectively, as additional markers of increased carbohydrate utilization. The skeletal muscle glycogen content is increased in male HuR^m−/−^ mice relative to controls (Figure 5G). Despite gene expression showing no difference in levels of the *ldh* gene, which encodes for lactate dehydrogenase (Figure 4E), serum lactate levels were also increased in male HuR^m−/−^ mice relative to controls (Figure 5H). Although female HuR^m−/−^ mice show a trend toward increases in levels of each of these markers, they are not significantly different between HuR^m−/−^ females and controls, or between HuR^m−/−^ females and HuR^m−/−^ males. This reinforces that the removal of HuR from skeletal muscle results in several increased similarities between male and female mouse muscle.

### 3.5 Both palmitate and pyruvate oxidation are decreased in HuR^m−/−^ male mice relative to controls

**FIGURE 6:**
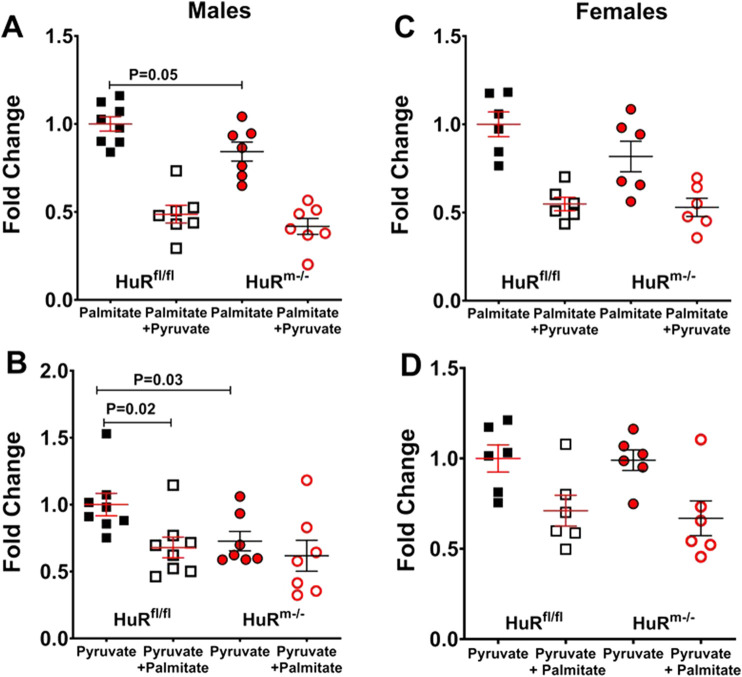
Not only palmitate but also pyruvate oxidation is decreased in male HuR^m−/−^ mice relative to controls. **(A, B)** Palmitate **(A)** or pyruvate **(B)** oxidation as measured by ^14^CO_2_ production in gastrocnemius homogenates from control (black) and HuR^m−/−^ (red) males. Palmitate oxidation was measured both in the absence (closed) and presence (open) of 1 mM pyruvate as an inhibitor. **(C, D)** Palmitate **(C)** or pyruvate **(D)** oxidation as measured by ^14^CO_2_ production in gastrocnemius homogenates from control (black) and HuR^m−/−^ (red) females. Pyruvate oxidation was measured both in the absence (closed) and presence (open) of 200 µM palmitate as an inhibitor. N = 6–8; *p < 0.05.

Our previous results have indicated that not only are mRNA transcripts of proteins involved in fat oxidation decreased in HuR^m−/−^ male skeletal muscle (Stone et al., 2021) but also mRNA associated with oxidative phosphorylation proteins is decreased (Mynatt et al., 2019). We therefore sought to test whether pyruvate oxidation was also decreased, which would implicate decreased mitochondrial function following glycolysis. Relative to controls, male HuR^m−/−^ mice show significant decreases in base oxidation of both palmitate and pyruvate (Figures 6A, B). Substrate competition designs were used for each, and the results showed that pyruvate effectively inhibited palmitate oxidation similarly in both genotypes; however, the ability of palmitate to serve as a competing substrate against pyruvate was less robust in HuR^m−/−^ males than in floxed controls (Figures 6A, B). Female HuR^m−/−^ mice show no differences in levels of maximal pyruvate or palmitate oxidation relative to controls, or in the shift toward oxidation of the alternate substrate in either assay (Figures 6C, D). These results provide further evidence that the removal of HuR from skeletal muscle results in the decreased utilization of mitochondrial metabolic substrates in a sex-specific manner.

## 4 Discussion

The degree to which mammals can switch between different substrates as cellular fuel has profound implications for the development and treatment of metabolic disease. Decreased HuR function in skeletal muscle is associated with decreased metabolic flexibility (Mynatt et al., 2019; Stone et al., 2021), and HuR removal from male mouse skeletal muscle correlates with increased fat mass, resulting in hallmarks of insulin resistance. This is a sex-specific phenomenon, with female HuR^m−/−^ mice not suffering the metabolic decrease associated with fat mass gain (Stone et al., 2021).

In the present study, we demonstrate that weight-matched male HuR^m−/−^ mice show several physiological indications of enhanced glucose utilization. These mice have greater glucose uptake, specifically in muscle tissues known to have a higher proportion of type 2 fibers in HuR^m−/−^ males relative to controls (Schiaffino and Reggiani, 2011). Male HuR^m−/−^ mice have been shown to have an increased prevalence of type 1 skeletal muscle fibers relative to controls (Janice Sanchez et al., 2019), which exhibit higher glucose uptake than type 2 fibers (Bocek et al., 1966). Our observed increase in glucose uptake in tissues such as gastrocnemius and EDL may thus be related to this shift. As increased uptake is seen only in males; it is worth noting that several studies report higher ratios of type 1/type 2 skeletal muscle fibers in female mammals (Haizlip et al., 2015; Nuzzo, 2023; Nuzzo, 2024). Our results together with these and other reports (Janice Sanchez et al., 2019) thus suggest that HuR removal results in increased similarity between male and female skeletal muscle.

Transcriptomic signatures reinforce this greater similarity between the sexes following the removal of HuR from skeletal muscle. Although female control mice show an increase in glycolysis pathway transcripts in skeletal muscle relative to males, this is ablated in HuR^m−/−^ animals. Our data suggest that this may be due to an increase in the expression of several glycolytic genes in male HuR^m−/−^ animals relative to controls. HuR may thus be involved not only in increasing fat metabolism but also in downregulating the glycolytic pathway.

Several additional pieces of evidence suggest enhanced glucose usage in male mice following the removal of HuR from skeletal muscle, including higher RER in HuR^m−/−^ males and increased glycogen storage. It is also noteworthy that HuR^m−/−^ males show an increase in serum lactate, which is associated with decreased metabolic flexibility and could indicate a tendency toward lactate metabolism due to decreases in mitochondrial oxidative capacity (San-Millan and Brooks, 2018).

Despite some increased similarities between male and female skeletal muscle lacking HuR, males and females show a different metabolic response. Male but not female HuR^m−/−^ mice have a decreased ability to oxidize not only palmitate but also pyruvate, which is the end product of glycolysis. This may indicate that although skeletal muscle signatures become more similar between the sexes following HuR removal, circulating factors within female animals may contribute to the differences in metabolic processing within the muscle. Indeed, serum levels of both adiponectin and estrogen are elevated in females relative to males and are known to enhance lipid oxidation in the periphery (Campbell and Febbraio, 2001; Sparks et al., 2009a; Sparks et al., 2009b). Further investigation of the role of these hormones in the metabolic differences between HuR^m−/−^ males and females will therefore be of interest in future studies.

Male HuR^m−/−^ mice showing decreased oxidation of both palmitate and pyruvate together with increased serum lactate levels relative to controls could also indicate decreased mitochondrial function. We have previously shown changes in mitochondrial signatures in male HuR^m−/−^ mice, including decreased oxidative phosphorylation and fat oxidation gene expression (Mynatt et al., 2019; Stone et al., 2021). HuR^m−/−^ mice also have increased mitochondrial copy number, and increases in Pgc1 (alpha), the mitochondrial biogenesis factor and Ppar coactivator Pgc1α (Stone et al., 2021; Janice Sanchez et al., 2019). This correlates with our present result indicating increased activation of Ppar signaling pathways in HuR mice. Although increased mitochondrial DNA can indicate increased function (Hood et al., 2000; Memon et al., 2021), it can also reflect mitochondrial defects (Castellani et al., 2020; Filograna et al., 2021). Our results showing decreases in oxidation of both palmitate and pyruvate thus encourage further investigation of mitochondrial integrity *via* morphological and respirometric analyses.

Finally, the introduction of pyruvate as an inhibitor of palmitate oxidation results in a much greater inhibition than when palmitate is used to inhibit pyruvate oxidation. Therefore, whereas fat oxidation is readily inhibited by a carbohydrate substrate, the reverse is not so, again suggesting a preference for carbohydrates in male skeletal muscle lacking HuR. The collection of these results thus shows a favorability for glucose in male HuR^m−/−^ muscle relative to controls, which is not the case for females. Given that several results reported here suggest greater ambiguity within skeletal muscle between male and female HuR^m−/−^ mice than between sexes within control mice, future studies aimed at understanding how HuR promotes mitochondrial oxidation are essential to fully elucidate its role in regulating metabolic flexibility in a sex-specific manner.

## Data Availability

The datasets presented in this study can be found in online repositories. The names of the repository/repositories and accession number(s) can be found at: The transcriptomics data presented in the study are deposited in the GEO repository, accession number GSE266882.

## References

[B1] Agrawal A. Balci H. Hanspers K. Coort S. L. Martens M. Slenter D. N. (2024). WikiPathways 2024: next generation pathway database. Nucleic Acids Res. 52 (D1), D679–D689. 10.1093/nar/gkad960 37941138 PMC10767877

[B2] Begaye B. Vinales K. L. Hollstein T. Ando T. Walter M. Bogardus C. (2020). Impaired metabolic flexibility to high-fat overfeeding predicts future weight gain in healthy adults. Diabetes 69 (2), 181–192. 10.2337/db19-0719 31712321 PMC6971489

[B3] Bocek R. M. Peterson R. D. Beatty C. H. (1966). Glycogen metabolism in red and white muscle. Am. J. Physiol. 210 (5), 1101–1107. 10.1152/ajplegacy.1966.210.5.1101 5947256

[B4] Bothe G. W. Haspel J. A. Smith C. L. Wiener H. H. Burden S. J. (2000). Selective expression of Cre recombinase in skeletal muscle fibers. Genesis 26 (2), 165–166. 10.1002/(sici)1526-968x(200002)26:2<165::aid-gene22>3.3.co;2-6 10686620

[B5] Campbell S. E. Febbraio M. A. (2001). Effect of ovarian hormones on mitochondrial enzyme activity in the fat oxidation pathway of skeletal muscle. Am. J. Physiol. Endocrinol. Metab. 281 (4), E803–E808. 10.1152/ajpendo.2001.281.4.E803 11551858

[B6] Castellani C. A. Longchamps R. J. Sun J. Guallar E. Arking D. E. (2020). Thinking outside the nucleus: mitochondrial DNA copy number in health and disease. Mitochondrion 53, 214–223. 10.1016/j.mito.2020.06.004 32544465 PMC7375936

[B7] Christofides A. Konstantinidou E. Jani C. Boussiotis V. A. (2021). The role of peroxisome proliferator-activated receptors (PPAR) in immune responses. Metabolism 114, 154338. 10.1016/j.metabol.2020.154338 32791172 PMC7736084

[B8] Filograna R. Mennuni M. Alsina D. Larsson N. G. (2021). Mitochondrial DNA copy number in human disease: the more the better? FEBS Lett. 595 (8), 976–1002. 10.1002/1873-3468.14021 33314045 PMC8247411

[B9] Ghosh M. Aguila H. L. Michaud J. Ai Y. Wu M. T. Hemmes A. (2009). Essential role of the RNA-binding protein HuR in progenitor cell survival in mice. J. Clin. Invest. 119 (12), 3530–3543. 10.1172/JCI38263 19884656 PMC2786787

[B10] Goodpaster B. H. Kelley D. E. (2002). Skeletal muscle triglyceride: marker or mediator of obesity-induced insulin resistance in type 2 diabetes mellitus? Curr. Diab Rep. 2 (3), 216–222. 10.1007/s11892-002-0086-2 12643176

[B11] Goodpaster B. H. Sparks L. M. (2017). Metabolic flexibility in health and disease. Cell Metab. 25 (5), 1027–1036. 10.1016/j.cmet.2017.04.015 28467922 PMC5513193

[B12] Griss J. Viteri G. Sidiropoulos K. Nguyen V. Fabregat A. Hermjakob H. (2020). ReactomeGSA - efficient multi-omics comparative pathway analysis. Mol. Cell Proteomics 19 (12), 2115–2124. 10.1074/mcp.TIR120.002155 32907876 PMC7710148

[B13] Haizlip K. M. Harrison B. C. Leinwand L. A. (2015). Sex-based differences in skeletal muscle kinetics and fiber-type composition. Physiol. (Bethesda) 30 (1), 30–39. 10.1152/physiol.00024.2014 PMC4285578 25559153

[B14] Heckmann L. H. Sorensen P. B. Krogh P. H. Sorensen J. G. (2011). NORMA-Gene: a simple and robust method for qPCR normalization based on target gene data. BMC Bioinforma. 12, 250. 10.1186/1471-2105-12-250 PMC3223928 21693017

[B15] Hom F. G. Goodner C. J. Berrie M. A. (1984). A [3H]2-deoxyglucose method for comparing rates of glucose metabolism and insulin responses among rat tissues *in vivo*. Validation of the model and the absence of an insulin effect on brain. Diabetes 33 (2), 141–152. 10.2337/diab.33.2.141 6363168

[B16] Hood D. A. Takahashi M. Connor M. K. Freyssenet D. (2000). Assembly of the cellular powerhouse: current issues in muscle mitochondrial biogenesis. Exerc Sport Sci. Rev. 28 (2), 68–73. 10902088

[B17] Janice Sanchez B. Tremblay A. K. Leduc-Gaudet J. P. Hall D. T. Kovacs E. Ma J. F. (2019). Depletion of HuR in murine skeletal muscle enhances exercise endurance and prevents cancer-induced muscle atrophy. Nat. Commun. 10 (1), 4171. 10.1038/s41467-019-12186-6 31519904 PMC6744452

[B18] Jung S. M. Sanchez-Gurmaches J. Guertin D. A. (2019). Brown adipose tissue development and metabolism. Handb. Exp. Pharmacol. 251, 3–36. 10.1007/164_2018_168 30203328 PMC7330484

[B19] Lam Y. Y. Redman L. M. Smith S. R. Bray G. A. Greenway F. L. Johannsen D. (2014). Determinants of sedentary 24-h energy expenditure: equations for energy prescription and adjustment in a respiratory chamber. Am. J. Clin. Nutr. 99 (4), 834–842. 10.3945/ajcn.113.079566 24500151 PMC3953881

[B20] Liberzon A. Subramanian A. Pinchback R. Thorvaldsdottir H. Tamayo P. Mesirov J. P. (2011). Molecular signatures database (MSigDB) 3.0. Bioinformatics 27 (12), 1739–1740. 10.1093/bioinformatics/btr260 21546393 PMC3106198

[B21] Memon A. A. Sundquist J. Hedelius A. Palmer K. Wang X. Sundquist K. (2021). Association of mitochondrial DNA copy number with prevalent and incident type 2 diabetes in women: a population-based follow-up study. Sci. Rep. 11 (1), 4608. 10.1038/s41598-021-84132-w 33633270 PMC7907271

[B22] Mootha V. K. Lindgren C. M. Eriksson K. F. Subramanian A. Sihag S. Lehar J. (2003). PGC-1alpha-responsive genes involved in oxidative phosphorylation are coordinately downregulated in human diabetes. Nat. Genet. 34 (3), 267–273. 10.1038/ng1180 12808457

[B23] Mynatt R. L. Noland R. C. Elks C. M. Vandanmagsar B. Bayless D. S. Stone A. C. (2019). The RNA binding protein HuR influences skeletal muscle metabolic flexibility in rodents and humans. Metabolism 97, 40–49. 10.1016/j.metabol.2019.05.010 31129047 PMC6624076

[B24] Nguyen N. T. Lindsey M. L. Jin Y. F. (2015). Systems analysis of gene ontology and biological pathways involved in post-myocardial infarction responses. BMC Genomics 16 (Suppl. 7), S18. 10.1186/1471-2164-16-S7-S18 26100218 PMC4474415

[B25] Nuzzo J. L. (2023). Narrative review of sex differences in muscle strength, endurance, activation, size, fiber type, and strength training participation rates, preferences, motivations, injuries, and neuromuscular adaptations. J. Strength Cond. Res. 37 (2), 494–536. 10.1519/JSC.0000000000004329 36696264

[B26] Nuzzo J. L. (2024). Sex differences in skeletal muscle fiber types: a meta-analysis. Clin. Anat. 37 (1), 81–91. 10.1002/ca.24091 37424380

[B27] O’Connell G. C. Treadway M. B. Petrone A. B. Tennant C. S. Lucke-Wold N. Chantler P. D. (2017). Leukocyte dynamics influence reference gene stability in whole blood: data-driven qRT-PCR normalization is a robust alternative for measurement of transcriptional biomarkers. Lab. Med. 48 (4), 346–356. 10.1093/labmed/lmx035 29069468 PMC5907901

[B28] Reimand J. Isserlin R. Voisin V. Kucera M. Tannus-Lopes C. Rostamianfar A. (2019). Pathway enrichment analysis and visualization of omics data using g:Profiler, GSEA, Cytoscape and EnrichmentMap. Nat. Protoc. 14 (2), 482–517. 10.1038/s41596-018-0103-9 30664679 PMC6607905

[B29] Ritchie M. E. Phipson B. Wu D. Hu Y. Law C. W. Shi W. (2015). Limma powers differential expression analyses for RNA-sequencing and microarray studies. Nucleic Acids Res. 43 (7), e47. 10.1093/nar/gkv007 25605792 PMC4402510

[B30] Robinson M. D. McCarthy D. J. Smyth G. K. (2010). edgeR: a Bioconductor package for differential expression analysis of digital gene expression data. Bioinformatics 26 (1), 139–140. 10.1093/bioinformatics/btp616 19910308 PMC2796818

[B31] Samuel V. T. Shulman G. I. (2016). The pathogenesis of insulin resistance: integrating signaling pathways and substrate flux. J. Clin. Invest. 126 (1), 12–22. 10.1172/JCI77812 26727229 PMC4701542

[B32] San-Millan I. Brooks G. A. (2018). Assessment of metabolic flexibility by means of measuring blood lactate, fat, and carbohydrate oxidation responses to exercise in professional endurance athletes and less-fit individuals. Sports Med. 48 (2), 467–479. 10.1007/s40279-017-0751-x 28623613

[B33] Schiaffino S. Reggiani C. (2011). Fiber types in mammalian skeletal muscles. Physiol. Rev. 91 (4), 1447–1531. 10.1152/physrev.00031.2010 22013216

[B34] Sparks L. M. Pasarica M. Sereda O. deJonge L. Thomas S. Loggins H. (2009a). Effect of adipose tissue on the sexual dimorphism in metabolic flexibility. Metabolism 58 (11), 1564–1571. 10.1016/j.metabol.2009.05.008 19595383

[B35] Sparks L. M. Ukropcova B. Smith J. Pasarica M. Hymel D. Xie H. (2009b). Relation of adipose tissue to metabolic flexibility. Diabetes Res. Clin. Pract. 83 (1), 32–43. 10.1016/j.diabres.2008.09.052 19038471 PMC2749984

[B36] Stone A. C. Noland R. C. Mynatt R. L. Velasquez S. E. Bayless D. S. Ravussin E. (2021). Female mice are protected from metabolic decline associated with lack of skeletal muscle HuR. Biol. (Basel) 10 (6), 543. 10.3390/biology10060543 PMC8233974 34204316

[B37] Storlien L. Oakes N. D. Kelley D. E. (2004). Metabolic flexibility. Proc. Nutr. Soc. 63 (2), 363–368. 10.1079/PNS2004349 15294056

[B38] Warfel J. D. Vandanmagsar B. Wicks S. E. Zhang J. Noland R. C. Mynatt R. L. (2017). A low fat diet ameliorates pathology but retains beneficial effects associated with CPT1b knockout in skeletal muscle. PLoS One 12 (12), e0188850. 10.1371/journal.pone.0188850 29240830 PMC5730174

