# Supplementary material for: Male mouse skeletal muscle lacking HuR shows enhanced glucose disposal at a young age
Source: Front Physiol. 2025 Feb 19;15:1468369. doi: 10.3389/fphys.2024.1468369 (PMC11880248; doi:10.3389/fphys.2024.1468369)

Supplementary Table 1. Primers used for qPCR analysis of genes from *mus musculus*.

Gene Symbol	Ref. Seq. ID	Protein Product	Forward (5'-3')	Reverse (5'-3')
Ldha	NM_001136069.2	Lactate Dehydrogenase	TGTGGCAGACTTGGCTGAGA	CTGAGGAAGACATCCTCATTGATTC
Ppib	NM_011149.2	Cyclophilin B (Control)	GGAGATGGCACAGGAGGAAA	CGTAGTGCTTCAGTTTGAAGTTCTCA
Hk2	NM_013820.4	Hexokinase	AAGGTTGACCAGTATCTCTACCA	AGGAACTCGCCATGTTCTGTC
Gpi	NM_008155.4	Glucose Phosphate Isomerase	TCACTGGATGGACCAGCACT	ATCTTGGTGCCTTGGTGGAT
Pfk	NM_001163487.1	Phosphofructokinase	CTATGATGCTTCAGCTGGGT	CTGTGATCTTCCCGTCTTTC
Aldoa	NM_007438.5	Aldolase	CGTAGGCAGCATGGCCAAAA	CACCCTTGTCAACCTTGATG
Tpi	NM_009415.3	Triose Phosphate Isomerase	ATCAGATGAGCTGATTGGCC	GTACTTCCTGTGCCTGCTGA
Gapdh	NM_001411840.1	Glyceraldehyde-3-Phosphate Dehydrogenase	AACGGATTTGGCCGTATTGG	GACTGTGGTCATGAGCCCTT
Pgk	NM_008828.3	Phosphoglycerate Kinase	CTGCTGGGCAAGGATGTTCT	ACACCCACCATGGAGCTATG
Pgm	NM_018870.3	Phosphoglycerate Mutase	GAGATGCTGGCTATGAATTT	ACCTGCGATCCTTGCTGATG
Eno1	NM_023119.3	Enolase	TGTGCCTGCCTTTAATGTGA	AGCTCCAGGGCCTCATTGTT
Elavl1	NM_010485	HuR	GGGATAAAGTAGCAGGACACAGCTT	GGGCGAGCATATGACACCTTAATGG
Pkm	NM_011099.4	Pyruvate Kinase	AAGGAGAAAGGCGCTGACTT	GATCTCATCAAACCTGCGGA

Supplementary Figure 1: Expression of *Elavl1* mRNA is Brown adipose tissue (BAT) and Gastrocnemius muscle (Gastroc) of male and female HuR$^{fl/fl}$ (black) and HuR$^{m-/-}$ (red) mice. N=4-5 Animals per group for BAT, and 8 animals per groups for Gastroc *P≤0.05.

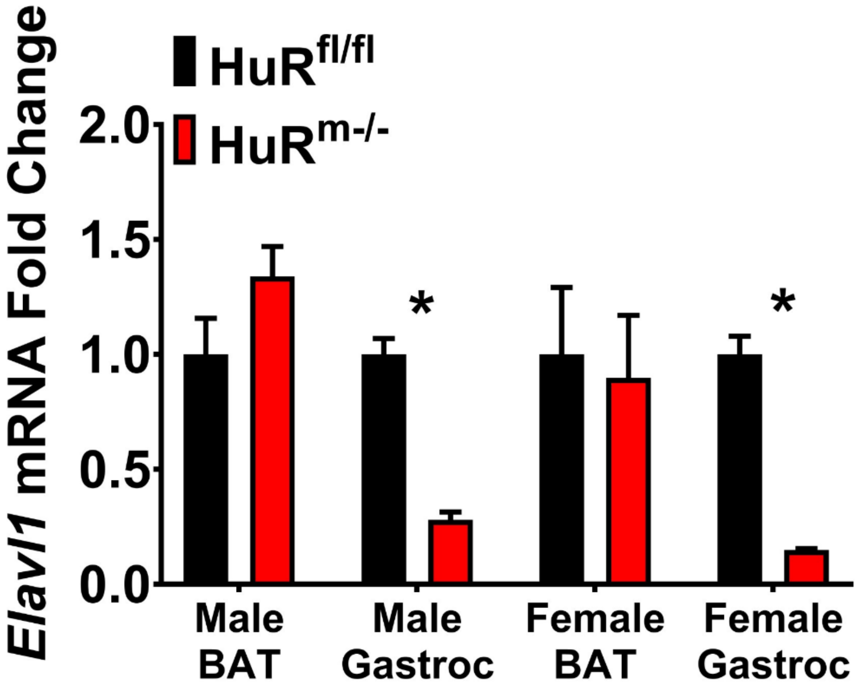

Supplementary Figure 2: Kyoto Encyclopedia of Genes and Genomes (KEGG) Top 60 pathways identified as differentially expressed through Gene Set Enrichment Analysis in comparison across all four groups (Male $HuR^{fl/fl}$, Male $HuR^{m-/-}$, Female $HuR^{fl/fl}$, Female $HuR^{m-/-}$).

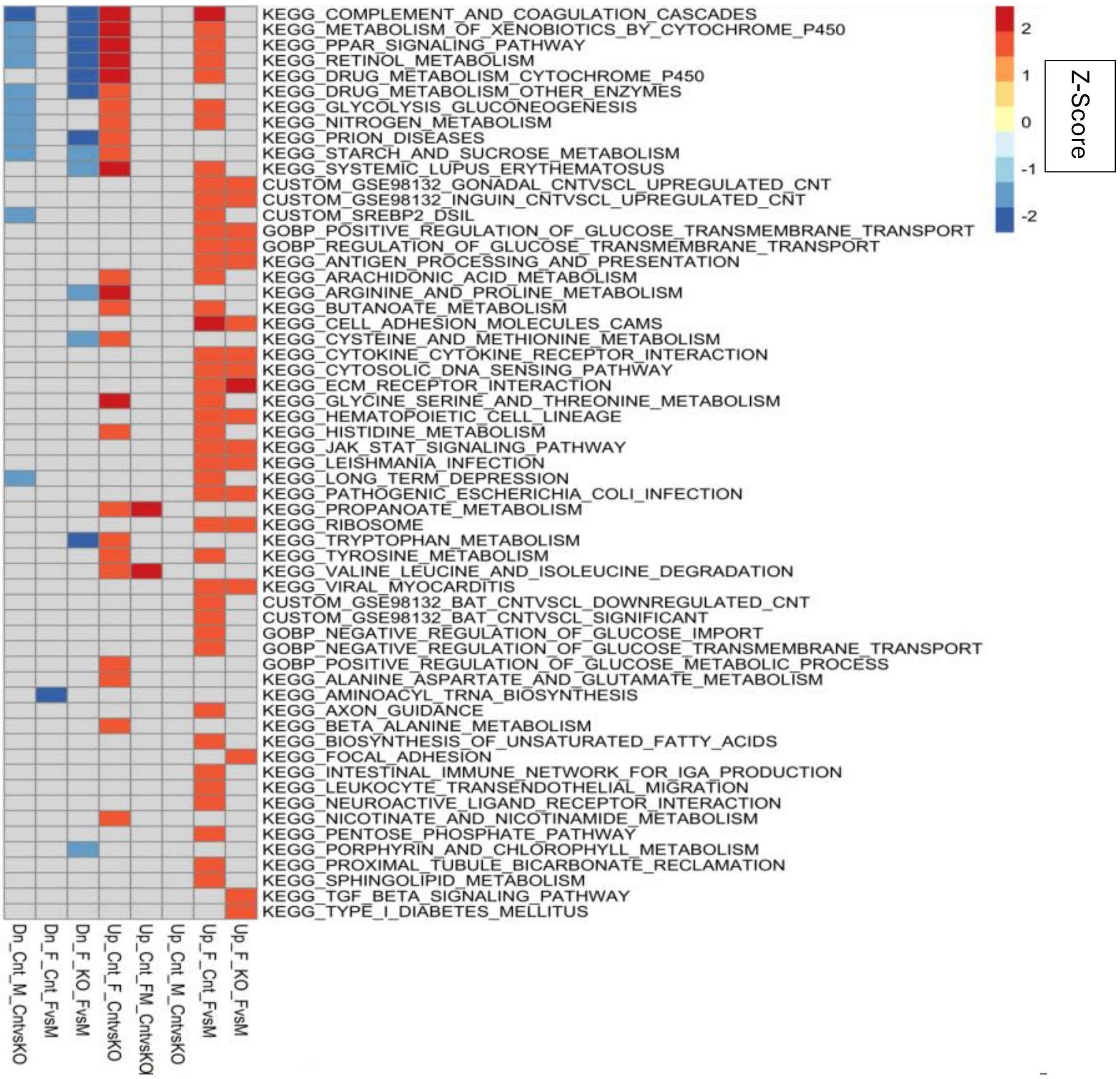

Supplementary Figure 3. Ppar Signaling pathway is differentially expressed in all comparisons (A-D) Kyoto Encyclopedia of Genes and Genomes (KEGG) Enrichment plots and Gene Set Enrichment Analyses showing differential expression of Ppar signaling pathway genes for each comparison of genotypes or sexes. (A) Male genotype comparison (Downregulated in Controls, FDR=0.03). (B) Female genotype comparison (Upregulated in Controls, FDR=0.001), (C) Control sex comparison (Upregulated in females, FDR=0.06), (D) HuR$^{m-/-}$ sex comparison (Downregulated in females, FDR=0.001). Blue boxes are drawn around significantly differentially expressed genes that are contributing to pathway significance N=5-7.

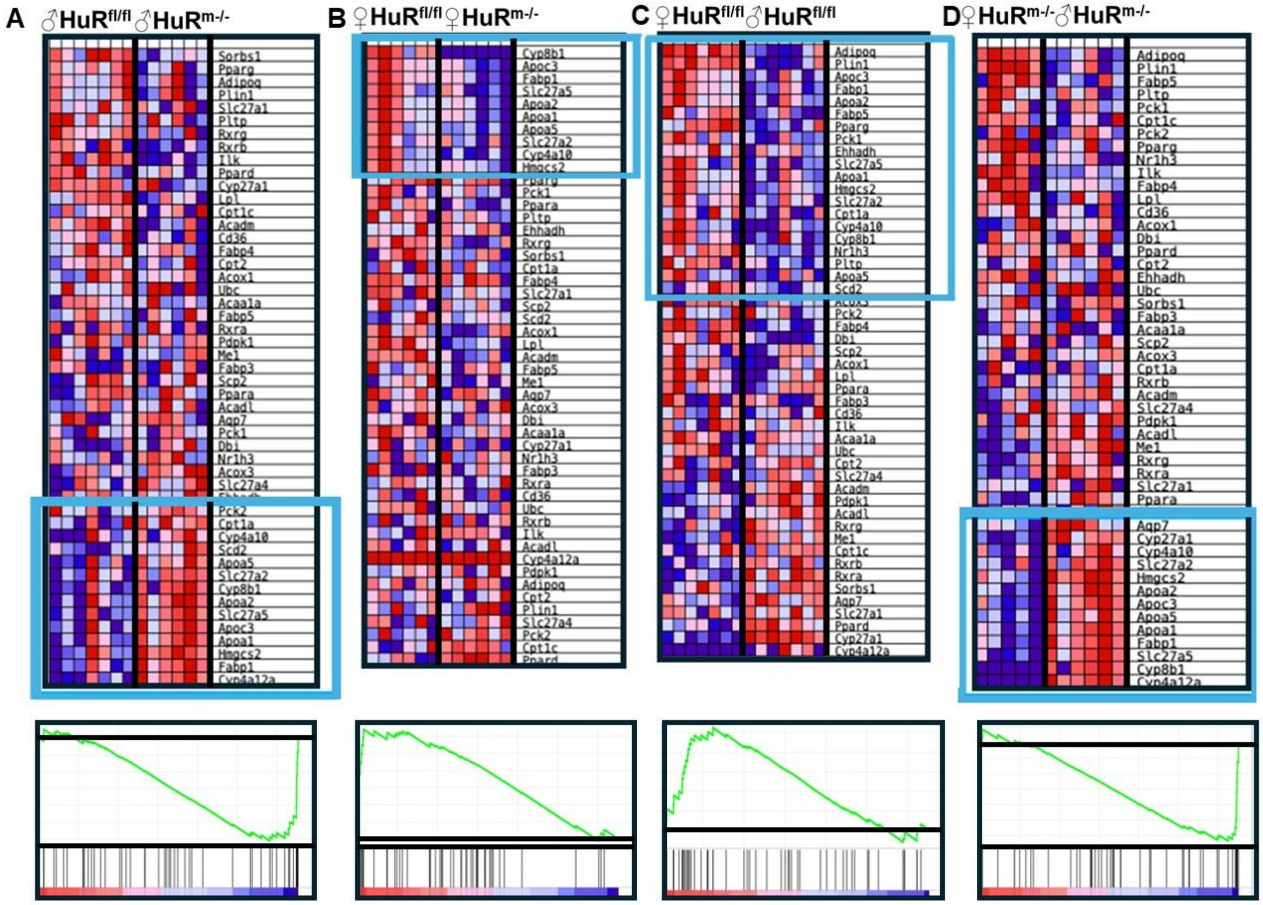

Supplementary Figure 4: Behavioral and indirect calorimetry measures for Male (A & C) and Female (B & D) $HuR^{fl/fl}$ (black squares) and $HuR^{m-/-}$ mice (red circles). Total activity (X+Y+Z beam breaks) is shown in A and B, and total food intake is shown in C and D. N=7-8 Animals per group, *P≤0.05.

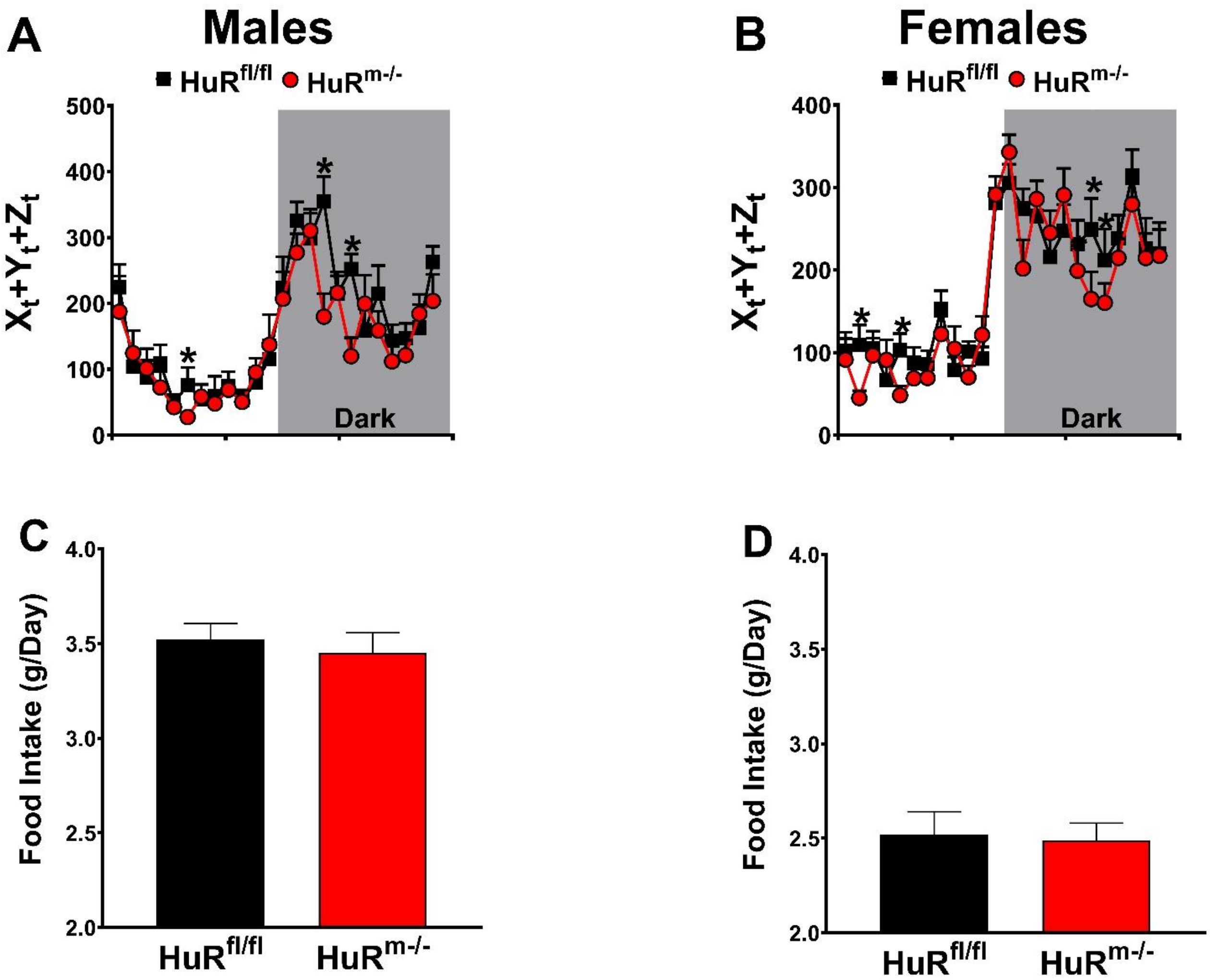

Supplement: Supplementary file 1 [file Table1.pdf]
